# Pathogenic characteristics of three genotype II porcine reproductive and respiratory syndrome viruses isolated from China

**DOI:** 10.1186/1743-422X-10-7

**Published:** 2013-01-03

**Authors:** Youjun Shang, Guangxiang Wang, Shuanghui Yin, Hong Tian, Ping Du, Jinyan Wu, Yan Chen, Shunli Yang, Ye Jin, Keshan Zhang, Zengjun Lu, Xiangtao Liu

**Affiliations:** 1 State Key Laboratory of Veterinary Etiological Biology, National Foot and Mouth Disease Reference Laboratory, Lanzhou Veterinary Research Institute, Chinese Academy of Agricultural Sciences, Xujiaping 1, Chengguan District, Lanzhou 730046, The People’s Republic of China

**Keywords:** Immune response, Pathogenic characteristics, Porcine reproductive and respiratory syndrome virus

## Abstract

**Background:**

We examined differences in pathogenicity in pigs from China that had been experimentally infected with porcine reproductive and respiratory syndrome virus (PRRSV).

**Methods:**

We compared pathogenic characteristics of a field isolate (GX-1/2008F), two PRRSV isolates (HN-1/2008, YN-1/2008) propagated in cells, and GX-1/2008F that had been propagated in cells (GX-1/2008). The clinical courses, along with humoral and cell-mediated responses, were monitored for 21 days post-infection (DPI). Animals were sacrificed and tissue samples used for gross pathological, histopathological and ultrastructure examination.

**Results:**

At 2–3 DPI, animals infected with cell-propagated viruses exhibited signs of coughing, anorexia and fever. However their rectal temperature did not exceed 40.5°C. Viremia was detectable as early as 3 DPI in animals infected with HN-1/2008 and YN-1/2008. Animals inoculated with GX-1/2008F displayed clinical signs at 6 DPI; the rectal temperature of two animals in this group exceeded 41.0°C, with viremia first detected at 7 DPI. Seroconversion for all challenged pigs, except those infected with GX-1/2008, was seen as early as 7 DPI. All of these pigs had fully seroconverted by 11 DPI. All animals challenged with GX-1/2008 remained seronegative until the end of the experiment. Innate immunity was inhibited, with levels of IFN-α and IL-1 not significantly different between control and infected animals. The cytokines IFN-γ and IL-6 transiently increased during acute infection. All virus strains caused gross lesions including multifocal interstitial pneumonia and hyperplasia of lymph nodes. Inflammation of the stomach and small intestine was also observed. Lesions in the group infected with GX-1/2008F were more serious than in other groups. Transmission electron microscopy revealed that alveolar macrophages, plasmacytes and lymphocytes had fractured cytomembranes, and hepatocytes had disrupted organelles and swollen mitochondria.

**Conclusions:**

The pathogenicity of the PRRSV field isolate became attenuated when propagated in MARC-145 cells. Tissue tropism of highly pathogenic strains prevailing in China was altered compared with classical PRRSV strains. The observed damage to immune cells and modulation of cytokine production could be mechanisms that PRRSV employs to evade host immune responses.

## Background

Porcine reproductive and respiratory syndrome (PRRS) is characterized by respiratory problems in young and mature pigs. PRRS is associated with reproductive failure in sows, late term abortion, stillbirth and increased pre-weaning mortality [1]. PRRS was first described in North America in 1987 [2,3], and in Europe in 1990 [4]. Porcine reproductive and respiratory syndrome virus (PRRSV) was first identified by Dutch researchers [5] in 1991 as the causative agent of PRRS. Almost every country worldwide has reported the occurrence of PRRS, with the exception of Switzerland, Sweden and Australia. PRRS has become one of the leading causes of economic losses in global swine operations. PRRSV is a single-stranded, positive-sense RNA virus belonging to the *Arteriviridae* family, and the order *Nidovirales*[3], which includes the *Equine Arteritis Virus*, *SimianHemorrhagic Fever Virus and Lactate Dehydrogenase-Elevating Virus*[6,7]. There are significant antigenic and pathogenic differences between the type I and II genotypes of PRRSV [8,9]. Following infection with either genotype, clinical signs are similar and result in respiratory disease and reproductive failure. In May 2006, a highly virulent form of PRRSV severely affected the pork industry of Jiangxi Province, China. Infections were characterized by high morbidity (50–100%) with high fever and mortality (20–100%) [10]. Since this initial outbreak, the disease has rapidly spread to most of the provinces in China, resulting in more than one million deaths [11]. Analysis of the genome sequence has indicated that highly pathogenic PRRSV (HP-PRRSV) contains an amino acid (aa) deletion at position 481 and a 29-aa deletion between positions 532–560 of the nsp2 gene [12]. Inactivated and live vaccines incorporating the NVDC-JXA1 strain of PRRSV have been used with some success; however, HP-PRRSV is still predominant in China. Many researchers have isolated HP-PRRSV strains, but experimental infections have shown that it is difficult to replicate the clinical signs of infection. There are some reports of PRRSV isolates losing their ability to infect susceptible animals after propagation in MARC-145 cells. Researchers have observed the occurrence of clinical signs, and even death in some studies resulting from experimental inoculations with viruses propagated in cells [10]. It would appear that there are as yet unknown differences among strains of PRRSV. To investigate these differences we isolated three genotype II PRRSV strains from tissue lesions and compared their pathogenic characteristics. Differences in pathogenic characteristics between a PRRSV strain from the field (GX-1/2008F), PRRSV strains propagated in cells (HN-1/2008, YN-1/2008) and the field isolate propagated in cells (GX-1/2008) were identified in this comparative study.

## Materials and methods

### Isolation and propagation of PRRSV strains

Virus strains used in this study were originally isolated from pigs suspected of harboring PRRSV. These pigs had a history of severe respiratory signs or abortion. Lung, spleen and lymph node samples were collected from Hunan, Yunnan and Guangxi Provinces in China. Collection and analysis of all field samples were done in Lanzhou Veterinary Research Institute, which is under the jurisdiction of Ministry of Agriculture, and is responsible for research on major epizootic. Samples were homogenized in Dulbecco’s modified Eagle’s medium (DMEM), and the tissue suspensions subjected to three cycles of freeze-thawing. Following centrifugation at 5,000 × g for 15 min, clarified samples were used in reverse-transcription polymerase chain reaction (RT-PCR) assays to detect PRRSV. The RT-PCR assays were conducted to detect open reading frame (ORF) 5 of PRRSV using specific primers (5′-CCT GAG ACC ATG AGG TGG G-3^′^ and 5^′^-TTT AGG GCA TAT ATC ATC ACT GG-3^′^). The DNA covering a putative discontinuous deletion of 30 aa in Nsp2 was detected with specific primers (5^′^-CGG TGG ACA CCA CCA CCC GGT-3^′^ and 5^′^-GTG GAT GAT GGC TTG AGC TGA G-3^′^). PRRSV-positive supernatants were passed through 0.22-μm filters and adsorbed to MARC-145 cell monolayers grown in DMEM containing 2% fetal bovine serum (FBS) for 2 h at 37°C/5% CO_2_. Cells were incubated at 37°C/5% CO_2_ and examined daily for cytopathic effects (CPE). When 75% CPE was observed, culture medium was freeze-thawed and cell lysates harvested and stored at −70°C. At passage 5, MARC-145 cells were screened using an immunoperoxidase monolayer assay (IPMA) [13]. We used specific polyclonal antibodies against PRRSV, classical swine fever virus (CSFV), porcine circovirus (PCV) and porcine parvovirus (PPV). GX-1/2008F was obtained by inoculating animals with a PRRSV-positive supernatant, which was confirmed to be free of CSFV, PCV and PPV. The lungs and lymph nodes of infected animals were collected at the end of the experiment.

### Virus titration

The titers of HN-1/2008, YN-1/2008 and GX-1/2008 were assessed at passage 5 when CPE was observed in MARC-145 cells, which had been seeded into 96-well plates 1 day prior to infection. Viral samples were serially diluted 10-fold in Dulbecco’s Minimal Essential Media (MEM) and added to wells (100 μL/well). Titration assays were conducted in quadruplicate. Cells were incubated for 7 days at 37°C/5% CO_2_, and the 50% tissue culture infective dose (TCID_50_) calculated according to the method of Karber.

### Animals and experimental design

Healthy weaning pigs (*n* = 25) that were 5–7 weeks old were selected from a PRRS-free farm. All animals were tested using routine serological assays and confirmed to be free of PRRSV, PPV, PCV and swine influenza virus (SIV) before they were used in experiments. Pigs were assigned to five groups based on weight. The five pigs in group A (pigs 1–5) were used as negative controls. Animals in group B (6–10) were inoculated with HN-1/2008, while those in groups C (11–15) and D (16–20) were inoculated with strains YN-1/2008 and GX-1/2008, respectively. The remaining five animals in group E (21–25) were inoculated with GX-1/2008F. Pigs in groups B, C and D were inoculated intranasally with 10^6^ TCID_50_ PRRSV. Pigs in group E were inoculated intranasally with a viral suspension from tissues containing 10^4^ TCID_50_ PRRSV. The negative control animals in group A received 6 mL of uninfected cell cultures. Experimental pigs were clinically examined for general behavior, feed intake, appetite, rectal temperature, respiratory rate (RR) and the presence or absence of clinical signs of respiratory disease or diarrhea each day from 0–21 days post-infection (DPI). To monitor viremia and evaluate levels of interleukin (IL)-1, IL-6, interferon (IFN)-α and IFN-γ, as well as seroconversion, blood samples were collected at 0, 3, 7, 11, 14, 17 and 21 DPI. All inoculated animals were sacrificed at 21 DPI for gross pathological, histopathological and ultrastructural examination. All animal procedures were in accordance with the Institutional Animal Care and Use Committee, and pigs were anesthetized before euthanizing.

### Detection of viremia

Serum samples were incubated on MARC-145 cells at 37°C/5% CO_2_ for 2 h. Serum was then aspirated and MEM added. Cells were incubated for 3 days at 37°C/5% CO_2_. Infection of inoculated cells was determined by IPMA. In addition to being used for viral isolation, serum samples were also examined by nested PCR (nPCR). Total RNA was extracted from 700 μL of serum using an RNeasy Mini Kit (Qiagen,Germany). A TaKaRa one-step RNA PCR Kit (AMV) was used to amplify ORF7 of PRRSV. Amplicons were used as templates in a subsequent reaction containing specific primers (5^′^-CGG AAT TCA TGC CAA ATA ACA ACG GCA AGC AGC-3^′^ and 5^′^-TAC TCG AGC TAT CAT GCT GAG GGT GAT GCT GTG-3^′^) that yielded a 372 bp amplicon. Cycling parameters for the one step RT-PCR were 50°C for 30 min and 94°C for 4 min, then 35 cycles of 94°C for 50 s, 56°C for 50 s and 72°C for 1 min. The cycling parameters for the second PCR were 94°C for 50 s, 58°C for 50 s, and 72°C for 50 s over 35 cycles.

### Humoral immune response

A commercially available enzyme-linked immunosorbent assay (ELISA) kit (HerdChek PRRS 2XR; IDEXX Laboratories, Westbrook, ME, USA) was used to detect and measure the concentration of PRRSV-specific antibodies. According to the manufacturer, sample to positive control (S/P) ratios greater than 0.4 were considered positive.

### Cytokine detection in serum

The concentrations of IL-1, IL-6, IFN-α and IFN-γ in serum samples were determined using commercial swine IL-1 and IFN-α ELISA kits (BioSource, CA USA) and porcine IL-6 and IFN-γ immunoassays (R&D Systems, Minneapolis, MN, USA). The minimum detectable concentration for IL-1 and IL-6 was 10 pg/mL, and 3.7 pg/mL for IFN-α. The IFN-γ assay had a sensitivity of 2 pg/mL. All samples were analyzed in duplicate.

### Detection of virus in tissue

At 21 DPI, all infected and control animals were euthanized and necropsied. Lung, spleen, kidney, liver, tonsil and lymphoid tissues were harvested and placed on ice. RT-PCR assays targeting transcripts encoding the M and N proteins were used to detect virus in these tissues. If RT-PCR assays were negative twice, then a nPCR was conducted to confirm the negative result. Specific primers used to detect the M protein gene were 5^′^-GAA CAA TGG GGT CGT CTT TAG A-3^′^ and 5^′^-TGG CAT ATT TGA CAA GGT TTA C-3^′^. Thermal cycling involved incubation at 50°C for 40 min, followed by an initial denaturation step at 94°C for 4 min, then 40 cycles of 94°C for 35 s, 46°C for 35 s, and 72°C for 90 s. All tissue samples were also analyzed by PCR for the presence of PPV, PCV and CSFV.

### Histological and ultrastructural examination

A part of each tissue sample was fixed in 4% paraformaldehyde and stained with hematoxylin and eosin (H&E) for histological examination. Another part of each tissue sample was fixed with 3% glutaraldehyde and examined using a transmission electron microscope (TEM).

### Statistical analysis

Differences in humoral response, cytokine production and viremia between different groups were determined with a one-way ANOVA and least significance difference (LSD). Differences were considered statistically significant when *P*-values were less than 0.05.

## Results

### Isolation and identification of PRRSV strains

CPEs were first apparent at passage 1 for each of the isolates. Infected MARC-145 cells became round and aggregated into clusters. On average, at 96 h post-infection (PI), 75% of cells were infected. Total RNA was extracted from cells at passage 4. PRRSV ORF5 and ORF7 were amplified and sequenced, and the sequence data submitted to GenBank (Accession numbers JQ308628, JQ308629 and JQ308630 for ORF7 of GX-2008, HN-1/2008 and YN-1/2008, respectively). Sequence analysis of ORF5 showed that the three isolates were closely related to HP-PRRSV, with 98.7–99.5% aa similarity and a unique deletion of 30 aa within the Nsp2-coding region. The IPMA results revealed that all three virus strains were only recognized by PRRSV antiserum. The TCID_50_ values for HN-1/2008, YN-1/2008 and GX-1/2008 after five passages in culture were 10^5.67^, 10^5.25^ and 10^5.5^, respectively. The TCID_50_ of GX-1/2008F was 10^3.25^.

### Clinical course and viremia

**Figure 1:**
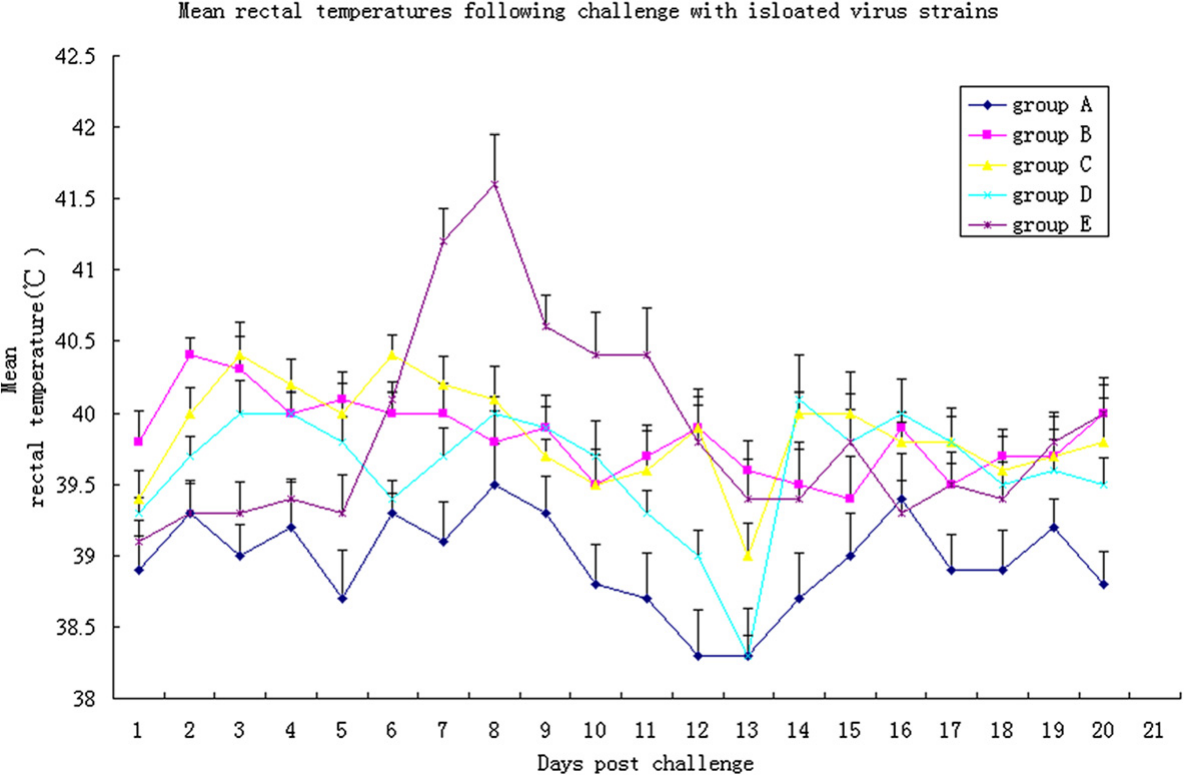
**Mean rectal temperatures following infection with HN-1/2008, YN-1/2008 and GX-1/2008.** Rectal temperatures equal to or above 40.0°C were defined as fever. Fever lasting 3 or more days was defined as illness. There were an average of 6–7 days of high fever for pigs in groups B, C and E, and 2 days of high fever for pigs in group D.

Control animals displayed no clinical signs throughout the study. No differences were observed in respiratory scores between control and inoculated animals until 2 DPI. From 3 DPI almost all inoculated animals, except those in group E, presented with cough, sneezing, anorexia and diarrhea. These signs were apparent until the animal experiment was terminated. Further, the skin of the hindquarter and abdomen were red, with black secretions from the eyes. Animals in group E displayed the clinical signs described above, but from 6 DPI onwards. The rectal temperature of all inoculated animals, except those in group E, were mildly elevated to 40°C at 2 or 3 DPI, persisting for 2–7 days, but did not exceed 40.5°C. The rectal temperature of animals challenged with GX-1/2008F was elevated to 40°C at 6 DPI, and exceeded 41°C for 5 days (Figure 1). No virus was detected in control animals throughout the study. All challenged animals were positive by nPCR at least once during the experiment, except for those in group D. Viremia was generally low with only one animal from groups B and C, and two from group E positive after a single round of RT-PCR. Viremia in the remaining pigs were detected by nPCR (Table 1). Additionally, virus could only be isolated from four pigs.

**Table 1:** Development of viremia in challenged animals

**DPI**						
		7	11	14		
Groups	3				17	21
Group A	0/5^†^	0/5	0/5	0/5	0/5	0/5
Group B	1/5	3/5	5/5	5/5	3/5	2/5
Group C	3/5	4/5	5/5	5/5	5/5	3/5
Group D	0/5	0/5	0/5	0/5	0/5	0/5
Group E	0/5	2/5	4/5	5/5	5/5	5/5

* Piglets in groups B, C and D were challenged with 10^6^ TCID_50_ of HN-1/2008,

YN-1/2008 PRRSV and GX-1/2008 cell-passaged viruses, respectively, and the piglets in group E were challenged with 10^4^TCID_50_ GX-1/2008 field virus.

†Serum samples were collected at 3, 7, 11, 14, 17 and 21 DPI, and viremia was tested by MARC-145 cell.

Inoculations and nPCR.

### Humoral immune response

**Figure 2:**
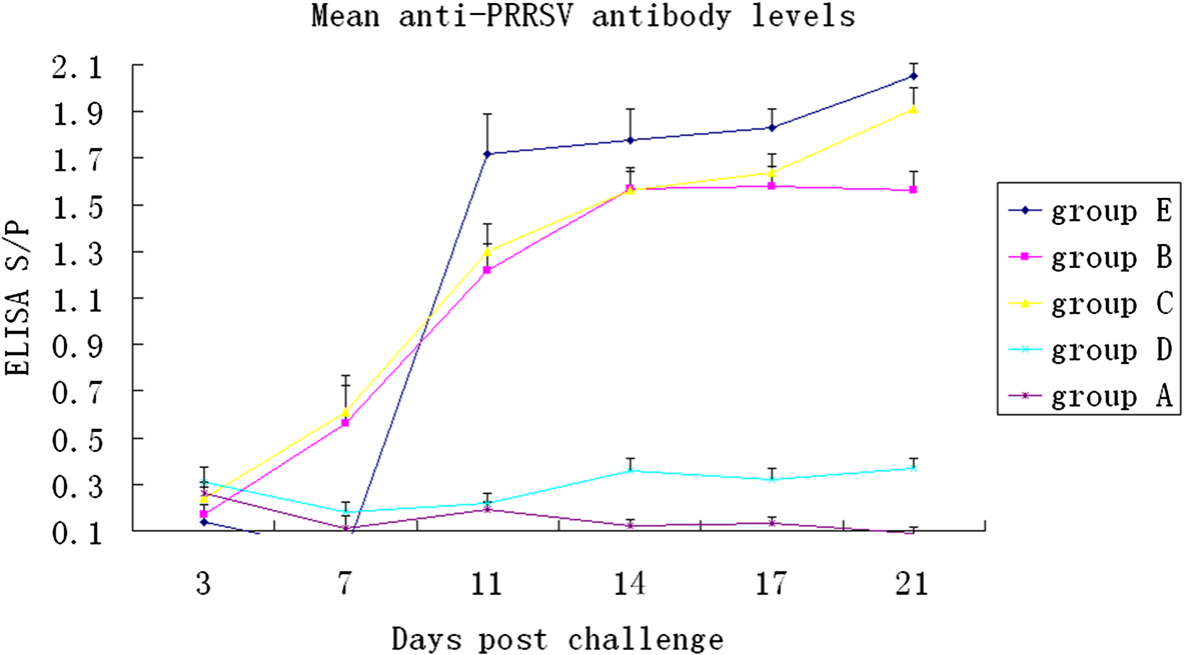
**Mean anti-PRRSV antibody levels.** Development of PRRSV specific antibodies was monitored throughout the experiment following infection and reported as S/P ratios. An S/P ratio greater than 0.4 was considered positive.

Animals challenged with GX-1/2008F had all seroconverted by 11 DPI. Two of the animals challenged with HN-1/2008 and YN-1/2008 seroconverted by 7 DPI and were clearly positive by 11 DPI. Animals infected with GX-1/2008 remained seronegative until the end of the experiment (Figure 2).

### Cytokine concentrations in serum

**Figure 3:**
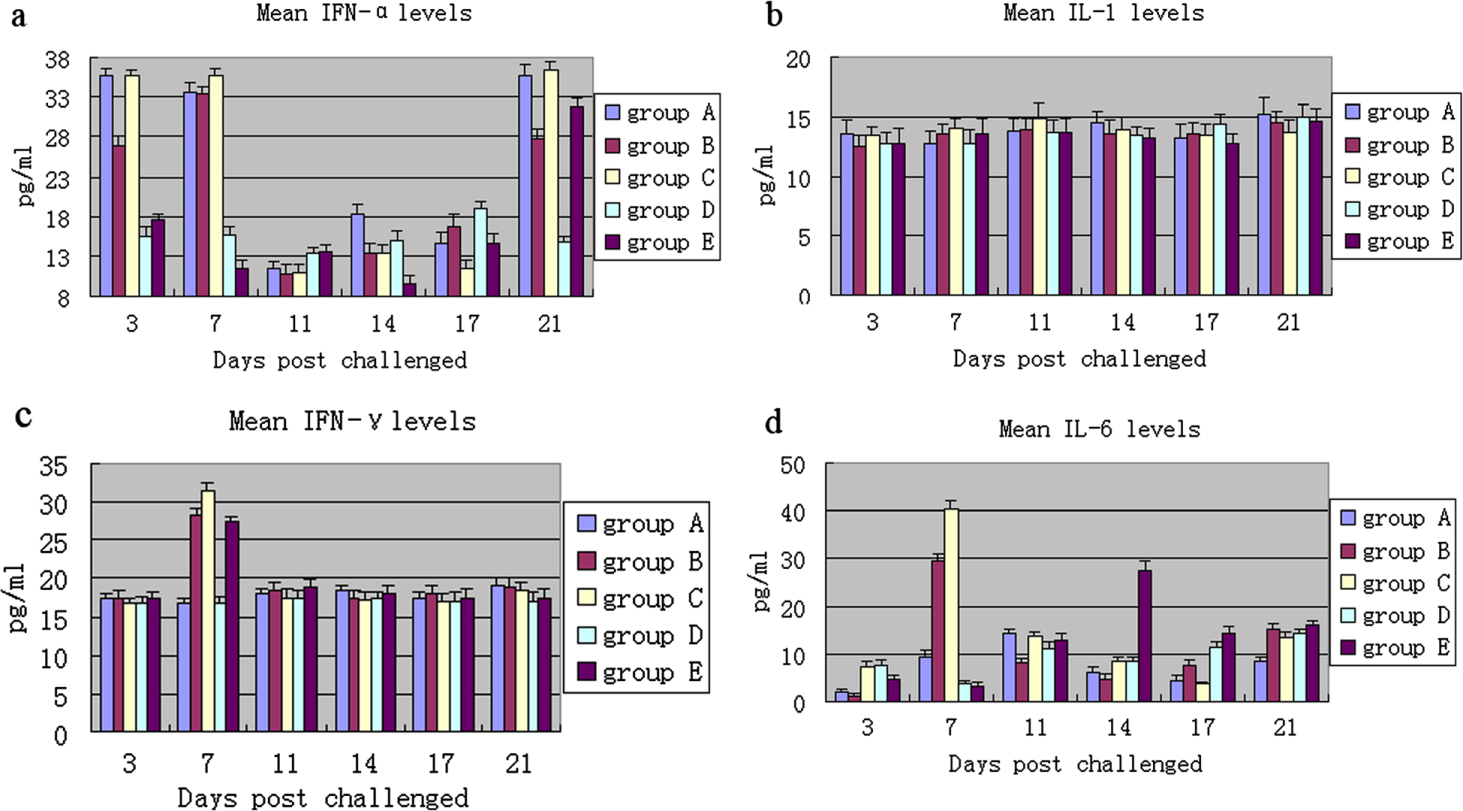
**Mean pro-inflammatory cytokines levels.** The development of pro-inflammatory cytokines was monitored throughout the experiment after infection. Mean levels for IFN-α (**a**), IL-1 (**b**), IFN-γ (**c**) and IL-6 (**d**) are presented.

Serum concentrations of IL-1 and IFN-α were not significantly different in infected animals compared with control animals (P > 0.05; Figure 3a and b). In contrast IL-6 and IFN-γ levels were enhanced at 7 DPI in groups B and C (P < 0.05; Figure 3c and d), and enhanced at 7 and 14 DPI in group E animals (*P* < 0.05). IL-6 and IFN-γ levels of group D animals were similar to that in control animals (*P* > 0.05), with IL-6 levels in group D slightly increased at 17 DPI (*P* > 0.05).

### Virus detection in tissues

Tissue samples from uninoculated pigs were negative for the presence of PRRSV. Tissues from animals in groups B, C and E were all positive for PRRSV after a single round of RT-PCR. The titer of PRRSV in group D was low. All group D animals were positive by nPCR; however, virus was only detected in the lung. All tissue samples from control and experimental animals were negative for the presence of PPV, PCV and CSFV.

### Pathological and histopathological examination

**Figure 4:**
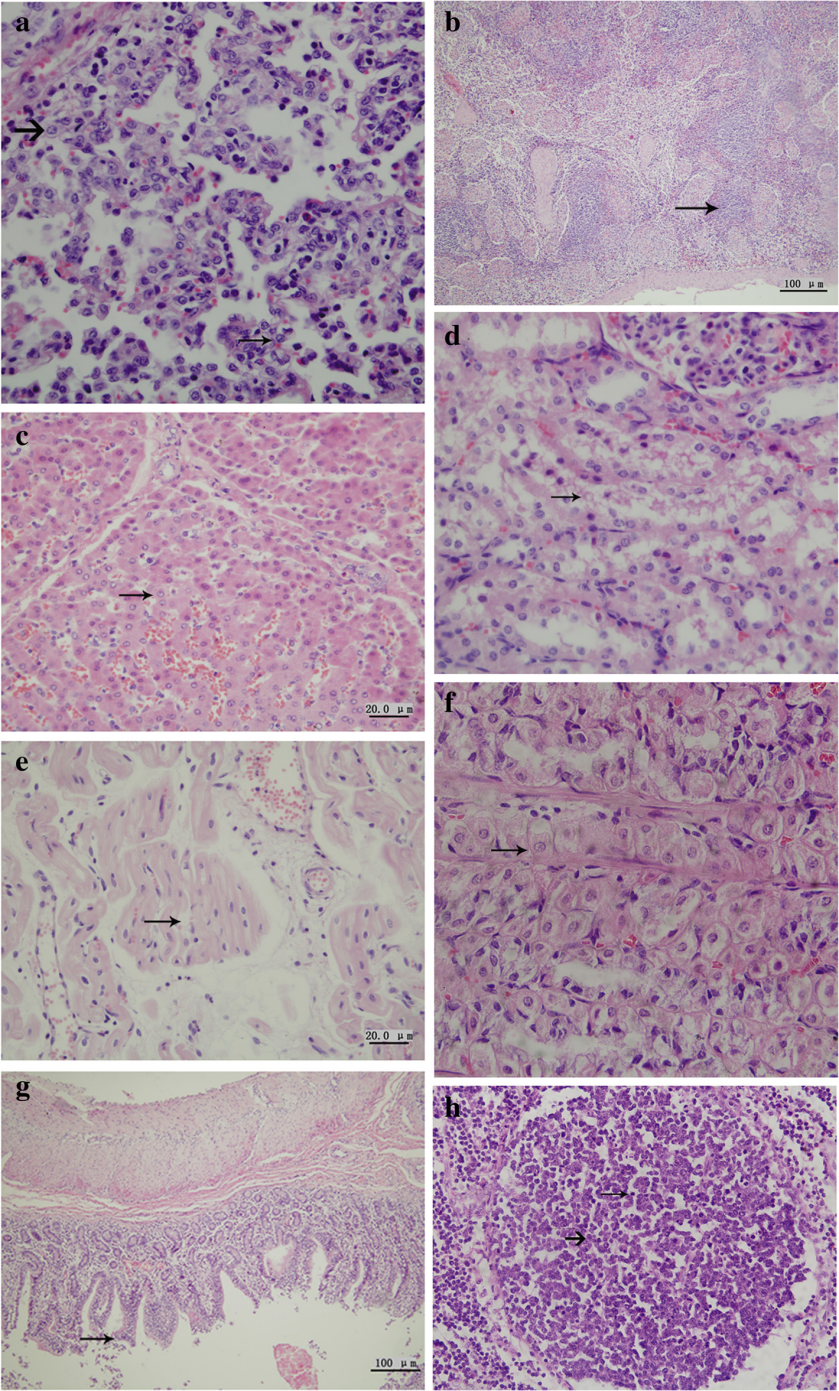
**Histological analysis.** Group E samples were examined by H&E staining at 21 DPI. (**a**) Alveolar septal thickening in macrophages indicated by a thick arrow. Hypertrophy of type II pneumocytes is indicated by a thin arrow. (**b**) Splenic corpuscles were diminished and the number of lymphocytes were decreased. (**c**) Swelling or destabilization in the structure of hepatocytes. (**d**) Kidney tubules were damaged. (**e**) The spaces between myocardial fibers were widened. (**f**) Gastric glands cells were swollen. (**g**) Necrosis and shedding of epithelial cells of the small intestine mucosa. (**h**) The number of lymphocytes were decreased (thin arrow) in mesenteric lymph nodes, with depletion of germinal centers (thick arrow).

**Figure 5:**
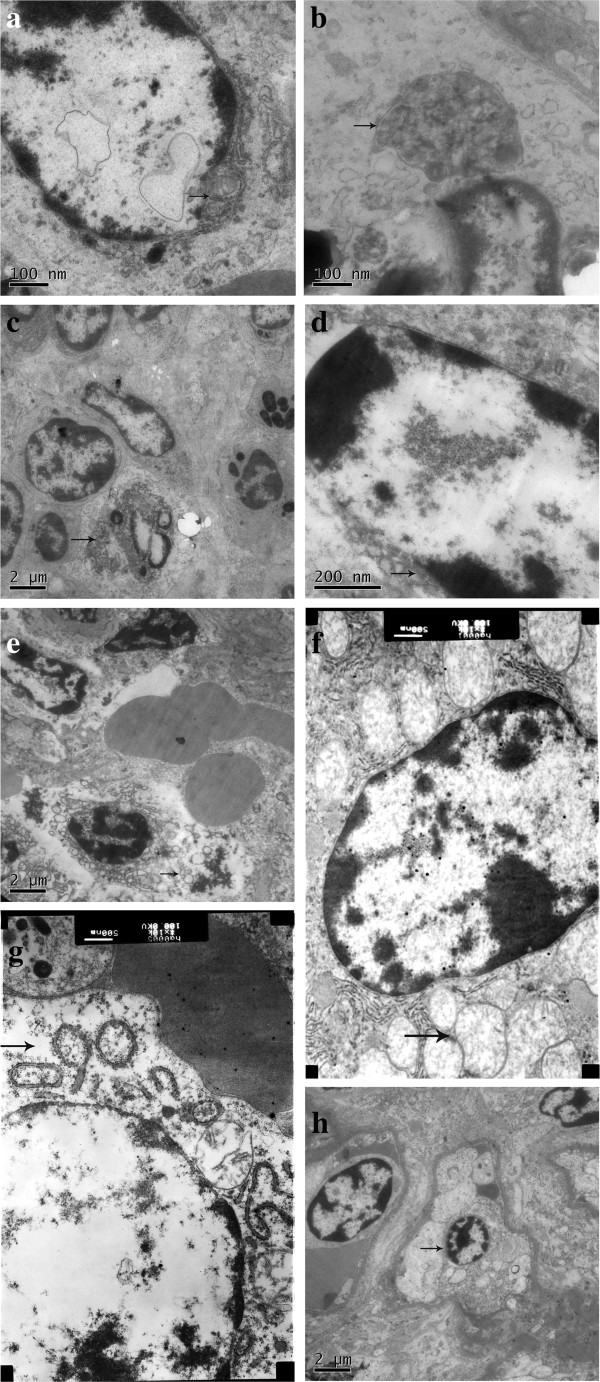
**TEM ultrastructural analysis.** Group E animals were examined at 21 DPI. (**a**) The nuclear envelope of alveolar macrophages lacked integrity. (**b**) Autophagosomes were seen in alveolar macrophages. (**c**) Lymphocytes in mesenteric lymph nodes were decreased. (**d**) The chromatin in splenic lymphocytes had migrated towards the margin of cells. (**e**) Numbers of splenic lymphocytes were decreased. (**f**) Mitochondria in liver cells were swollen, with the number of mitochondrial crista decreased or absent. (**g**) The majority of organelles in hepatocytes were decayed. (**h**) Nuclei of glomerular vascular endothelial cells were pyknotic.

Lesions were obvious and observed in all pigs from groups B–E. Lesions in group E animals were more severe than those in other groups. In group D animals, lesions were mainly found to occur in the lungs and lymph nodes. Gross pathological changes were characterized by interstitial pneumonia and systemic enlargement of inguinal, mesenteric, hilar and mandibular lymph nodes. Lungs were diffusely red, non-collapsing, firm and rubbery. The percent area of pathological change for groups B, C, D and E were approximately 8, 11, 3 and 15%, respectively. Hilar, mesenteric and inguinal lymph nodes of all piglets in group E were firm, hemorrhaged and enlarged. Most pigs in groups B and C also displayed lesions on hilar and mesenteric lymph nodes, with some hemorrhagic spots on the surface of hilar lymph nodes in most pigs from group D. Three pigs in group B presented with hemorrhage and ulceration in the stomach, but these pathological changes were not observed in other groups. The spleens of four pigs in group E were swollen, with red nodules on the surface. Very few animals in groups B and C displayed the same lesions. There were some white nodules in the lobus apicalis and lobus diaphragmaticus of most pigs in group B. One pig in group D and two in group E presented with white nodules in the lobus diaphragmaticus. Group B animals contained no heart lesions but inflammatory fluid was found in the pericardium. Ventricles of group C animals were congested, but there was no fluid in the pericardium. The ventricles of both groups D and E animals were also congested with inflammatory fluids in pericardia. The gross lesions for all groups are summarized in Table 2. Infected animals developed significant microscopic lung lesions compared with those in the control group. Interstitial pneumonia was characterized by a marked thickening of the alveolar septa by macrophages and occasionally hypertrophy of type II pneumocytes. Lesions observed in the spleen and lymph nodes were characterized by collapsed follicles, depletion of germinal centers and decreased lymphocyte counts. The space between myocardial fibers was widened. Mucosal epithelia of the small intestine had partially shed. Gastric gland cells appeared swollen. Microscopic examination revealed severe inflammation of the lung, heart, liver, kidney, intestines and stomach, which was characterized by infiltrating lymphocytes, macrophages and necrocytosis. Microscopic lesions observed in the groups are summarized in Figure 4. TEM examination revealed that the cytomembrane of cells, such as alveolar macrophages, type II alveolar cells and plasmacytes, were fractured. Chromatin in glomerular vascular endothelial cells and plasmacytes appeared at margins. Lesions observed in the lung were characterized by a decrease in the number of type I alveolar cells, and an increase in the number of type II alveolar cells. The number of alveolar macrophages remained the same. Lymphocyte proliferation was observed in hepatic blood sinuses. In hepatocytes, almost all organelles were dissolved; the rough endoplasmic reticulum appeared anomalous, mitochondria were swollen, and the number of mitochondrial crista were decreased or had completely disappeared. The number of glomerular mesangial cells and the mesangial matrix were increased. Some virus inclusion bodies and autophagosomes were observed in alveolar macrophages and lymphocytes of mesenteric lymph nodes. Ultrastructural changes due to viral infection are summarized in Figure 5.

**Table 2:** Histopathologic lesions in organs of piglets after challenge by different viruses

** Groups**	**Lung**		**Lymph node**							
					**Liver**	**Spleen**	**Kidney**	**Stomach**	**Intestine**	**Heart**
	++*	+	++	+						
Group A	0/5	0/5	0/5	0/5	0/5	0/5	0/5	0/5	0/5	0/5
Group B	2/5	3/5	5/5	0/5	3/5	2/5	2/5	3/5	2/5	2/5
Group C	4/5	1/5	4/5	1/5	0/5	1/5	3/5	0/5	3/5	2/5
Group D^†^	0/5	5/5	0/5	3/5	1/5	0/5	0/5	0/5	1/5	1/5
Group E	5/5	0/5	5/5	0/5	2/5	4/5	4/5	0/5	4/5	3/5

* Histological changes were scored as “+”. The number of “+” represents the severity of impairment.

^†^ In group D, lesions were mainly localized to the lung and lymph nodes. In groups B, lesions were detected in all tissues, groups E and C did not detected stomach lesions, and groups C also did not detected liver lesions.

## Discussion

The main goal of this study was to investigate the pathogenic characteristics of PRRSV field isolates. When PRRSV-positive supernatants of field strains were inoculated into MARC-145 cells, CPE appeared at the first passage, indicating that the isolated viruses were initially highly pathogenic in these cells. These isolates easily adapted to MARC-145 cells but were different from viruses isolated before 2005 [10], which had to be adapted to porcine alveolar macrophages (PAMs) for several passages before successful propagation in MARC-145 cells. While the isolates we examined in this study replicated as well as HP-PRRSV strains JXA1 and HUB1 isolated from 2006–2007, there were some differences in pathogenicity among HN-1/2008, YN-1/2008 and GX-1/2008. The times at which 75% CPE was observed for HN-1/2008, YN-1/2008 and GX-1/2008 were 84, 92 and 100 h, respectively. Phylogenetic trees based upon ORF5 and ORF7 indicated that these strains were all genotype II PRRSV, and clustered on the same branch as JXA1 and HUB1. Similarities among the protein homologs for ORF7 among these five strains were 100%. Similarities between highly pathogenic and two typical strains (CH-1a/2001 and VR-2332/2000) were 94.3%. This highlighted that highly pathogenic strains have become predominant among epidemic PRRSV strains in China.

To confirm the pathogenicity of HN-1/2008, YN-1/2008 and GX-1/2008 in susceptible animals, we inoculated piglets in groups B–D with 10^6^ TCID_50_ of HN-1/2008, YN-1/2008 and GX-1/2008 after five passages in cells. Piglets in group E were inoculated with 10^4^ TCID_50_ of GX-1/2008F. Approximately 2–6 days post-challenge, all inoculated animals presented with clinical signs, but no animals died. The pathogenic characteristics of the isolates in this study were consistent with those of JXA1and HUB1. However, mortality rates were low, suggesting that the virulence of highly pathogenic strains prevailing in China have abated. Additionally, the pathogenicity of the field sample was significantly different to the isolates that were passaged in cell cultures. Although clinical signs induced by HN-1/2008 and YN-1/2008 presented earlier than those of the field isolate, the pathological changes that were observed in all infected piglets were similar. The extent of these changes, along with the maximal rectal temperatures caused by HN-1/2008 and YN-1/2008, were not as apparent as those caused by GX-1/2008F.

The clinical signs and pathological changes induced by GX-1/2008 and GX-1/2008F were very different, even though the dose of the isolate passaged in cells was higher than that of the field sample. This indicates that virulence and pathogenicity of the field isolate became attenuated after passage in MARC-145 cells. There have been some reports regarding the loss of infectivity of isolates in susceptible animals after propagation in MARC-145 cells. The reason for this phenomenon has not been fully elucidated. Zuckermann [14] developed a PAM cell line, ZMAC-3, which was shown to efficiently support the replication of a number of PRRSV isolates. They injected piglets with an equivalent dose of the Prime Pac vaccine grown in either ZMAC-3 or MARC-145 cells. After 4 weeks, all vaccinated animals were challenged with an “atypical PRRS abortion storm” virus isolate (NADC-20). It was found that the virus used in vaccines and grown in ZMAC-3 cells was significantly more effective than that derived from MARC-145 cells at reducing viremia and eliminating virus from the lungs. The effectiveness of a PRRS modified-live virus vaccine is not only determined by its genetic similarity to the challenge virus, but is also influenced by how it is produced. At present, three PRRSV receptors have been identified on PAMs: heparan sulfate (HS); sialoadhesin (Sn, also known as CD169); and CD163. Early attachment of PRRSV is mediated mainly *via* interaction with HS, while Sn is required for PRRSV attachment and internalization. Although HS is not essential for Sn-mediated internalization, it has been shown to enhance internalization and infection [15]. It is possible that binding Sn is a necessary first step in a pathway leading to uncoating and release of viral RNA into the cytoplasm [16]. HS, CD163, and CD151 have been identified or proposed as cellular receptors, co-receptors or mediators for in vitro PRRSV infection in MARC-145 cells. Because MARC-145 cells do not express Sn, it has been suggested that simian vimentin can play the role of Sn in that cell line [17]. We concluded that the attenuation, or loss of pathogenicity, of field isolates following passage in MARC-145 cells may be related to changes in conformation of PRRSV proteins responsible for attachment, infection or propagation.

The main gross lesions caused by PRRSV generally consist of a multifocal interstitial pneumonia with mottled areas and hyperplasia of lymph nodes [18]. The pathological changes caused by the highly pathogenic strains in this study were similar to those previously reported. Pathological changes were found in almost in every tissue examined in this study. Previous studies of piglets with acute viremia after PRRSV infection did not report inflammation in the stomach or the small intestine [19], although Gao *et al.*[20] discovered that piglets challenged with the VR-2332 strain exhibited hyperemia of the stomach and small intestine, but no epithelial shedding was observed.

The tropism of highly pathogenic strains examined in this study had altered compared with those previously studied. PAMs are the preferred target cell of PRRSV infection. In this study, a comparison of the number of PAMs between control and infected animals showed no decrease in their numbers. However, we did observe some virus inclusion bodies and autophagosomes in those cells. As reported by Xiao *et al.*[21], we found that only a small proportion of PAMs were infected. Xiao *et al.* found that less than 2% of lung macrophages were PRRSV-positive during an acute infection, without causing a significant decline in local macrophage frequency in tissues. They deduced that this phenomenon may be due to the rapid replacement of destroyed macrophages; however, the majority of macrophages might not be permissive to PRRSV infection. Therefore, the elimination of permissive cells would limit the extent of infection. In our work, the presence of autophagosomes in alveolar macrophages indicated that infected macrophages were eliminated by normal macrophages, strongly supporting the findings of Xiao *et al.* Additionally, the numbers of types І and II alveolar cells were decreased and increased, respectively, but were all damaged. Type І alveolar cells reside in the respiratory tissue. Type II alveolar cells are the precursors of type І alveolar cells. We propose that the number of type II alveolar cells increase when type І alveolar cells are damaged. Respiratory obstruction occurred when types I and II alveolar cells were damaged, weakening the function of the respiratory system. Damage to cellular organelles such as the rough endoplasmic reticulum and mitochondria in plasmacytes and lymphocytes showed that infection with PRRSV induced immunosuppression.

Using ELISA, development of specific antibodies in groups B and C animals could be observed as early as 7 DPI. Pigs in group E were seropositive at 11 DPI. Although piglets in group D displayed clinical signs, and analysis by nPCR showed that tissues were PRRSV-positive, these animals remained seronegative throughout the experiment. It is unclear why group D animals did not seroconvert, but has been previously reported by Hermann *et al.*[22]. They found that virus could be isolated from an animal exposed to 10^5.2^ TCID_50_/ml at 7, 14 and 21 DPI, even though it was seronegative at all sampling points (0, 4, 14 and 21 DPI). They were unable to detect other clinical diseases or other agents of disease. Tests for the detection of PPV, PCV and CSFV antigens were also negative. No animals in group D had detectable viremia. However, the majority of piglets in other groups were viremic, but virus could only be detected after using a highly-sensitive nPCR assay. These results suggest that seroconversion and clinical manifestations were related to the presence of virus in the blood.

Innate immunity plays a crucial role in the defense against viral infections. It is characterized by rapid induction upon infection, and by lacking memory and specificity [23]. The response of animals infected with RNA viruses involves the production of type I interferons (IFN-α and/or -β), and can often be accompanied by secretion of tumor necrosis factors (TNFs) and IL-1 [24]. In our study, the levels of IFN-α and IL-1 in all infected and control animals were not significantly different. The ability of the three isolates to induce IFN-α was similar, suggesting that PRRSV infection inhibited or suppressed the innate immune responses in these animals. IFN-γ plays a key role in cell-mediated immune responses against a variety of cytopathic viral infections in animals. Several reports have shown that IFN-γ can inhibit PRRSV infection [25]. We investigated the kinetics of PRRSV-specific IFN-γ levels in serum samples during an acute infection. An increase in the expression of IFN-γ levels was observed at 7 DPI after infection with GX-1/2008F, HN-1/2008 and YN-1/2008. Elevation in IFN-γ levels was not observed for group D animals. IFN-γ is known to protect macrophages against PRRSV replication in vitro [26]. However, viral replication still occurred following the induction of IFN-γ. We concluded that in our animal study the temporary IFN-γ response was not efficient to eliminate PRRSV.

IL-1β is known to induce IL-6 production. It has been demonstrated that the appearance of IL-1 and −6 are accompanied with the onset of clinical respiratory disease and increased body temperature [27]. In the present study, IL-6 levels in groups B, C, and E were elevated at 7, 7, and 14 DPI, respectively. At these time points, pneumonia and other clinical signs, especially fever, were apparent. Our results showed that the changes in expression for these cytokines likely reflect the level of pathology induced by the virus.

## Conclusions

HN-1/2008 and YN-1/2008 propagated in MARC-145 cells and GX-1/2008F induced pathogenic disease in susceptible animals. The virulence of GX-1/2008F was greatest, while the virulence and pathogenicity of GX-1/2008 was attenuated. Tissue tropism of highly pathogenic strains that are prevalent in China were altered, with these strains becoming less virulent. The observed damage to immune cells, disruption of organelles and the modulation of cytokine production are likely to be mechanisms used by PRRSV to evade the host immune response.

## Competing interests

None of the authors had any financial or personal relationships that could inappropriately influence or bias the content of our manuscript.

## Authors’ contributions

YS: study design, coordination and preparation of the manuscript. SYi and HT: pathological and histopathological examination. GW and PD: isolation and propagation of PRRSV strains. JW,YC and SYa: detection of viremia and cytokines. YJ, KZ and ZL: animal experiments. XL: project leader. All authors read and approved the final manuscript.
